# Anti-seizure potential of J4, an equilibrative nucleoside transporter 1 inhibitor, in a mouse model of tuberous sclerosis complex in response to pentylenetetrazol

**DOI:** 10.1186/s13578-025-01518-3

**Published:** 2026-01-03

**Authors:** Christine Chin-jung Hsieh, Nai-Kuei Huang, Szu-Yu Tung, Wei-Xuan Lin, Hsin-Hui Wang, Thu Thi Anh Nguyen, Yijuang Chern, Yi-Chao Lee

**Affiliations:** 1 Biomedical Translation Research Center, Academia Sinica, Taipei, Taiwan; 2 Taipei Neuroscience Institute, Taipei Medical University, Taipei, Taiwan; 3 Ph.D. Program in Medical Neuroscience, College of Medical Science and Technology, Taipei Medical University, No. 250, Wu- Hsin St, Taipei, 11031 Taiwan; 4 National Research Institute of Chinese Medicine, Ministry of Health and Welfare, Taipei, Taiwan; 5 School of Biotechnology, International University, Ho Chi Minh City, Vietnam; 6 International Master Program in Medical Neuroscience, College of Medical Science and Technology, Taipei Medical University, Taipei, Taiwan; 7 Institute of Biomedical Sciences, Academia Sinica, Taipei, Taiwan; 8 Neuroscience Research Center, Taipei Medical University, Taipei, Taiwan

**Keywords:** Tuberous sclerosis complex, J4, Adenosine, Equilibrative nucleoside transporter 1, Epilepsy, Pentylenetetrazol (PTZ)-kindling model

## Abstract

Tuberous sclerosis complex (TSC) is a neurodevelopmental disorder with epileptic seizures caused by genetic mutations in either TSC1 or TSC2 gene. Multiple genetic, epigenetic, and environmental factors can affect the phenotypical outcomes of TSC individuals. Accumulating evidence has shown that the seizures occurred in early life may contribute to the epileptogenesis and aggravate the neurological setting and neuropsychiatric symptoms of TSC. Therefore, treatments targeting seizures and/or epileptogenesis have always been the main focus on TSC therapies. Current anti-epileptic drugs and mTOR inhibitors show some efficacy, yet up to one-third of TSC-epileptic individuals are classified as refractory epilepsy. Vigabatrin, which has been used as the first-line therapy for infantile spasms in TSC, has demonstrated to delay the onset and lower the overall incidence of seizures in infants with TSC when it was used as a preventive treatment. Recently, because of its efficacy, cannabidiol, which targets adenosine signaling pathway, has been approved by the U.S. FDA for the treatment of TSC-associated epilepsy, suggesting an anti-epilepsy strategy other than mTOR inhibition is also plausible for TSC. To this end, we sought for a preventative treatment of an adenosine pathway-targeted therapeutic strategy. In this study, we pretreated *Tsc2*^+/–^ mice with J4, an equilibrative nucleoside transporter 1 inhibitor, before the initiation of kindling epileptogenesis driven by the repetitive PTZ induction paradigm. We found that J4 reduced the seizure behavior severity in *Tsc2*^+/–^ mice, as well as decreased mossy fiber sprouting resulted from the aberrant neurogenesis upon PTZ injurious insults. We also found that J4 increased the expression of GluR2, inhibited the astrogliosis and microgliosis, and eventually prevented the neuronal cell loss due to the excitotoxicity. The present study provides a new alternative therapeutic concept for pretreating TSC-related epilepsy before the epileptogenesis process.

**Supplementary Information:**

The online version contains supplementary material available at 10.1186/s13578-025-01518-3.

## Introduction

Tuberous sclerosis complex (TSC) is a hereditary disease caused by the gene mutation in either TSC1 or TSC2, which encodes hamartin and tuberin, respectively. These two proteins function together to repress mTOR signaling. Deficiency in either protein will result in hyperactivation of mTOR, thereby affecting various organs in the whole body. The brain pathology is the leading cause of morbidity and mortality. Up to 90% of the patients exhibit neurological symptoms include seizures, such as infantile spasm or status epilepticus, and other cognitive, psychiatric or behavioral deficits.

Epilepsy is commonly found in TSC patients. According to the International multicenter ‘TuberOus Sclerosis registry to increase disease Awareness (TOSCA)’, 84% of TSC patients had epilepsy, and 39% of them had infantile spasms (IS) and 68% had focal seizures [1]. Vigabatrin (VGB) is recommended as the first-line anti-epileptic drug (AED) for IS and focal seizures in TSC infant below 2 years of age [2, 3]. Despite that VGB has a great efficacy on infantile spasms (76.3% was controlled with the treatment), there was still 38% of patients with focal seizures that were not controlled with the treatment [1]. Many traditional AEDs are used in combination and/or as replacement therapy for refractory TSC-associated focal seizures [3]. Overall, 38% to 62.5% of TSC patients with epilepsy are classified to be refractory to AEDs [4]. In addition, mTOR inhibitors such as sirolimus (rapamycin) and everolimus have been approved by the U.S. FDA as an adjunctive treatment for drug-resistant TSC-associated epilepsy based on the results of the clinical study [5]. However, only 40% of TSC refractory epileptic patients responded to the everolimus treatment [6].

Recently, the U.S. FDA has approved cannabidiol (Epidiolex^®^) for the treatment of TSC-associated seizures in patients one year of age and older, in addition to two rare serious forms of epilepsy which are previously approved, Lennox-Gastaut syndrome and Dravet syndrome [7, 8], . Cannabidiol (Epidiolex^®^) has been shown to be effective in treating seizures in TSC [9]. One of the proposed mechanisms of action of cannabidiol is the modulation of adenosine-mediated signaling [10] and its effect on the ENTs [11]. In addition, cannabidiol has been shown to exert its immunosuppressive effects through enhancing adenosine level via the inhibition of ENT [12]. These studies emphasized the potency of adenosine-modulating ENT1 inhibitors for the treatment of TSC-associated epilepsy.

Adenosine augmentation has been considered one of the effective mechanisms in treating intractable epilepsy [13–15]. Several studies have shown that during epileptogenesis, maladaptive overexpression of adenosine kinase (ADK), which regulates intracellular adenosine level by converting adenosine into 5’-adenosine-monophosphate (AMP), leads to adenosine deficiency and subsequently epilepsy [16–18]. Therefore, many groups have demonstrated that in different rodent models of kindling, epilepsy progression can be prevented through adenosine augmentation [16, 19–21]. The role of adenosine in epilepsy has been thoroughly studied. The adenosine binds to both pre- and post-synaptic A_1_Rs to exerts its inhibitory effects on neuronal excitability through 3 mechanisms [15]. First, activation of pre-synaptic A_1_Rs inhibits excitatory neurotransmitter release and block calcium channels [22, 23]. Second, it acts on the post-synaptic terminal, where potassium and calcium channel dynamics are controlled through signaling G_i/o_ proteins, promoting cell membrane hyperpolarization [24]. Third, activation of A_1_Rs modulates GABAergic transmission by interacting GABA_A_ receptors and inhibiting GABA uptake by astrocytes [25].

Two types of therapeutic strategies for treating epileptogenesis through elevating cellular adenosine levels have been investigated, one is ADK inhibitors, and the other is equilibrative nucleoside transporter 1 (ENT1) inhibitors. The development of ADK inhibitors have previously gained attraction [14, 26]. However, due to the toxicological findings of the compounds, the preclinical and clinical trials were halted [26, 27]. On the other hand, ENT1 inhibitors have been studied in treating seizures through elevating adenosine as well. ENT proteins, encoded by the solute carrier family 29 (SLC29A), include four members in human, namely hENT1, hENT2, hENT3 and hENT4. All 4 members transport adenosine but differed in their transport capabilities for other nucleosides and nucleobases [28]. Both hENT1 and hENT2 control the adenosine flux bidirectionally across the cell membrane of astrocytes and neurons [29, 30]. A past study has shown that specifically inhibiting ENT1 by nitrobenzylthioinosine (NBTI) may reduce the seizure severity in a pilocarpine-induced rat epileptic seizure model [31]. However, direct targeting A_1_Rs as a therapeutic strategy for epilepsy remains a significant problem due to that activation of A_1_Rs would cause adverse effects such as sedation, hypothermia, bradycardia and hypotension [32–34]. Novel pharmacological methods are needed to target the adenosine receptors to prevent these unwanted events [33, 34].

J4, which is an orally bioavailable, brain blood barrier-permeable adenosine analog, originally isolated from the traditional Chinese herb *Gastrodia elata* [35] and known to inhibit ENT1 with a Ki value of 50 nM and IC50 of 0.068 µM [36]. In this study of Lee et al., the administration of J4 into the hippocampus significantly raised the extracellular adenosine level, which exerts its effects primarily through four distinct G-protein-coupled receptors: A_1_, A_2A_, A_2B_, and A_3_ receptors [36]. It is also known to reduce the activation of both microglia and astrocytes, thereby preventing the excessive release of inflammatory cytokines such as TNF-α in a mouse model of tauopathy [37, 38]. Likewise, in a spinal cord injury (SCI) mouse model, inhibition of ENT1 by J4 led to reduced neuroinflammation and a recovery of motor functions [39]. In addition, it has been demonstrated to improve the seizure latency onset and survival rate in a repetitive low dose pentylenetetrazol (PTZ) kindling model [40]. In this present study, we therefore aim to elucidate the potential therapeutic effects of J4 on the seizure susceptibility of the *Tsc2*^+/–^ knockout mice evoked by PTZ.

## Materials and methods

### Animals

Animals used in this study were treated in accordance with guidelines of the University Committee on the Care and Use of Experimental Animals of Taipei Medical University (Taipei, Taiwan). Mice were housed in an air-conditioned vivarium with free access to food and water and a 12/12-h light/dark cycle. Only male animals (7-week-old to 14-week-old) were used due to hormonal cycle variability in female mice. Research shows that seizure frequency and severity are affected by hormonal fluctuations during menstrual cycle or pregnancy [41]. The *Tsc2*^+/–^ knockout mouse model (B6;129S4-Tsc2tm1Djk/J) was purchased from Jackson laboratory (Bar Harbor, ME, USA).

### PTZ-induced seizures

Two types of pentylenetetrazol- (PTZ-) induced models were used. First, a PTZ kindling model using an every-other-day, low-dose PTZ administration schedule was used [42]. In brief, PTZ was prepared in sterile 0.9% (w/v) NaCl at a concentration of 3.5 mg/ml of PTZ on the day and injected intraperitoneally at a dose of 35 mg/kg and was injected every other day for a total of 21 injections when the mice were 7–8 weeks old (*N* = 8–9). Second, a modified PTZ kindling protocol was followed. To observe the seizure susceptibility and chronic seizures in *Tsc2*^+/–^ mice, a sub-convulsant dose of 40 mg/kg of PTZ was intraperitoneally injected every other day when the mice were 9–10 weeks old (*N* = 3–6). A total of 6 injections of PTZ were administered.

After PTZ injection, mice were placed in a clear observing cage for 30 min and video recorded for behavioral seizure scoring and seizure frequency quantification. The severity of seizures and scoring were based on published scoring criteria [42], with some modifications. The Racine scale was modified and described in brief as follows: 0, normal; 1, immobility and lying on belly; 2, head nodding, forelimbs or hindlimbs twitching; 3, myoclonic jerks, tail held up; 4, rearing, clonic seizures, falling on its side; 5, tonic-clonic seizure, wild jumping; 6, death. Mice were considered successfully kindled if they exhibited three consecutive Racine stage ≥ 3 seizures after 10 or more PTZ injections. Animals that did not reach a Racine stage of 3 after 10 or more injections were excluded from behavioral analyses.

### Drug treatments

For the WT PTZ kindling model, J4 treatment was given *ad libitum* in the drinking water at the dose of 0.06 mg/ml. The desired amount of J4 was dissolved in the drinking water containing 1% HPβCD, which served as the vehicle. The treatment started as the PTZ kindling procedures began, which was at 7 weeks of age, and continued throughout the whole PTZ kindling paradigm. The total treatment time was 7–8 weeks.

Vigabatrin (VGB) was prepared as follows. Vigabatrin (Sabril^®^, Sanofi-aventis) was provided in tablet form. The tablets (500 mg/each) were ground into a fine powder using a mortar and pestle, and the powder was dissolved in sterile drinking water, to achieve a final concentration of 3.5 mg/ml. The VGB-drinking water was given to the mice at 7 weeks of age, and continued throughout the whole PTZ kindling paradigm.

For the PTZ-induced seizures in *Tsc2*^+/–^ mice, two doses of J4 treatment was used in this study, 0.02 mg/ml and 0.06 mg/ml. The treatment started at the age of 7 weeks. The pretreatment of J4 duration was 2 weeks before the PTZ injections and continued during the PTZ kindling procedures. The total treatment time was 4 weeks. 0.02 mg/ml dose of J4 treatment was used based on its efficacy in tauopathy and AD models as previously described [36, 37]. A previous study showed that intraperitoneal administration of J4 at 10 mg/kg body weight reduced hindlimb extension in a seizure model [40]. Based on the daily water intake of mice under specific dietary conditions [43], 0.06 mg/ml in drinking water is approximately equivalent to 10.2 mg/kg body weight. Hence, 0.06 mg/ml dose was used herein to deliver exposures closer to intraperitoneal regimens used for decreasing seizure activity.

### Brain slice preparations and immunostaining

Mice were subjected to anesthesia with Zoletel^®^ (50 mg/kg; tiletamine hydrochloride and zolazepam hydrochloride) and Rompun^®^ (12 mg/kg; xylazine hydrochloride) before transcardial perfusion using 10% formalin and then decapitated. After extracted from the skull, the brains were post-fixed with 10% formalin, at 4 ℃ for overnight. Fixed brains were dehydrated in 30% sucrose in 0.5 M PB for 4 days prior to OCT embedding. Sections with 30 μm thickness were obtained using Leica CM1950 freezing microtome (Leica Biosystems, Wetzlar, Hesse, Germany).

To carry out the free-floating immunofluorescence staining, the slices were incubated with desired antibodies for overnight at 4℃. The following primary antibodies were used in this study, GFAP, Iba1, NeuN to visualize the astrocytes, microglia and neurons, respectively. After washing with PBS, the slices were incubated with the corresponding Alexa Fluor dye-tagged secondary antibodies at room temperature for 1.5 h. After washing by PBS 3 times, tissue slices were mounted onto the slide(s) and mounted with anti-fading mounting medium (Vector Laboratories, Burlingame, CA, USA) and cell nuclei were stained with Hoechst 33,258 (Sigma-Aldrich, Burlington, MA, USA). Images were acquired by Leica Stellaris 8 confocal fluorescent microscope (Leica Biosystems, Wetzlar, Hesse, Germany).

For Nissl staining, the brain slices were first mounted onto the slides. The slides with the tissues are placed in the cresyl violet acetate solution for 5 min. The slides were rinsed briefly with PBS, followed by dehydration in graded alcohols (i.e., 50%, 75%, and 95% alcohol). Finally, the slides were cleared in xylene and mounted with mounting medium. Images were acquired by TissueGnostics and visualized with TissueFAXS & HistoFAXS (TissueGnostics GmbH).

For the Fluoro-Jade C (FJC) staining, the brain slices were stained according to the manufacturer’s protocol (Cat.# TR-100-FJ, Biosensis Pty Ltd., Australia). In brief, the brain slices were mounted on gelatin-coated and dried at 50–60 ℃ oven overnight. The slides were first incubated in basic ethanol (80% ethanol:/NaOH) for 5 min, and then transferred to 70% ethanol for 2 min, followed by rinsing in distilled water for 2 min. The slides were then oxidized in 0.006% solution of potassium permanganate (KMnO_4_) for 10 min, followed by a rinse in distilled water for 2 min. The slides were transferred to FJC solution with DAPI for 10 min with gentle shaking covered with foil to protect from light. Subsequently, the slides were washed in distilled water for 3 times, 1 min each. The slides were dried and cleared by xylene for 5 min and cover-slipping with mounting media. The slides were visualized by Leica Stellaris 8 confocal fluorescent microscope (Leica Biosystems, Wetzlar, Hesse, Germany).

### Quantitative analysis

For quantitation of immunofluorescence staining, 3 random areas from the brain regions of interest were selected from 3 to 5 different animals (*N* = 3–5) from each group and analyzed. The area percentage of immunoreactivity of each antibody was determined by the “Measure” function of the Fiji/ImageJ software (NIH, Bethesda, MD, USA; https://imagej.net/Fiji). The images underwent background subtraction before measuring. The values presented were normalized to the control or WT mice. For quantitation of Nissl-stained images, 3 random equal areas of the desired brain region were selected from 3 to 5 different animals (*N* = 3–5) from each group and analyzed. The cell density of each image was determined by counting the number of cells in an area of 200 μm × 200 μm using the “Analyze Particles” function of the Fiji software and validated by a different experimenter.

### Microglia morphological analysis

Iba1 immunofluorescence staining was used for analyzing soma size, circularity, and Sholl and skeleton analyses were performed using Fiji plugins according to the protocol previously published [44, 45]. For each group, containing 3–4 animals, 10–15 microglial cells were randomly chosen with a total of 40 cells per group for soma assessment. For Sholl and skeleton analyses, 3–5 single cells were chosen with a total of 10 cells from each group were isolated, converted to binary images, pre-processed, skeletonized and analyzed.

### Statistical analysis

All statistical analyses were carried out using Prism 10.0 (GraphPad Software Inc., San Diego, CA, USA). For immunostaining image and Nissl-staining analyses, immuno-intensity and cell number were assessed using one-way ANOVA with appropriate *post-hoc* tests for multiple comparisons. The datasets were tested and confirmed for the normality using D’Agostino & Pearson, Anderson-Darling, and Kolmogorov-Smirnov tests. For the Racine score in PTZ-induced seizures, two-way ANOVA with appropriate *post-hoc* tests for multiple comparisons was used. For the average Racine score at the first injection, Kruskal-Wallis test with appropriate *post-hoc* tests for multiple comparisons was used.

## Results

### J4 Lowered seizure severity caused by PTZ-induced epileptic seizures

**Fig. 1:**
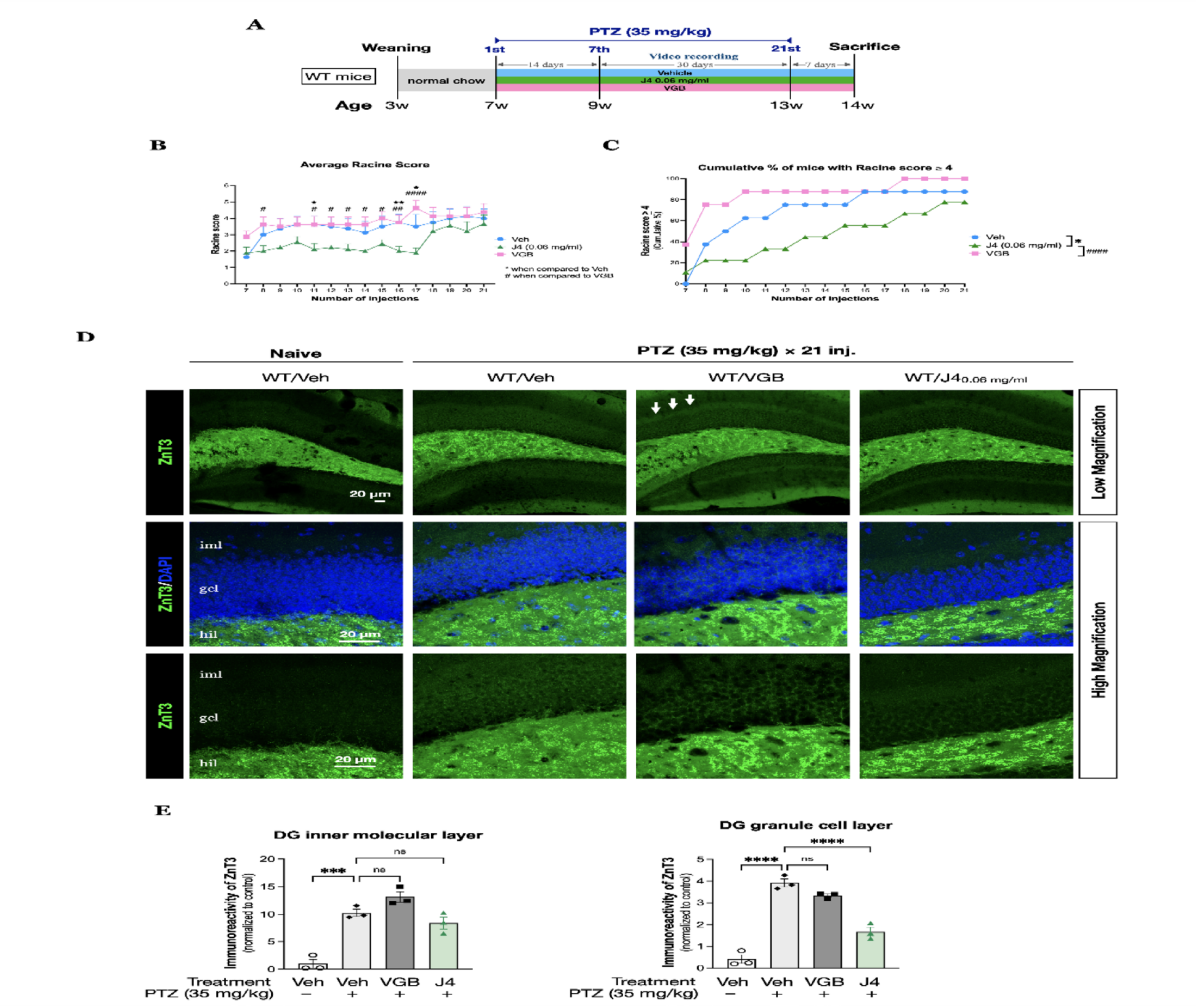
**A** The experimental procedural diagram of low-dose PTZ kindling model. **B** PTZ at a dose of 35 mg/kg was administered to PTZ-WT/Veh (*N* = 8), PTZ-WT/J4 (*N* = 9) and PTZ-WT/VGB (*N* = 8) as indicated. Racine score was determined starting from 7th injection to 21st injection for each group. Racine score was compared between the PTZ-WT/J4 group and the PTZ-WT/Veh group, and the statistical significance was indicated by the asterisks (*). Racine score was compared between PTZ-WT/J4 and the PTZ-WT/VGB, and the statistical significance was indicated by the number sign (#). **C** The cumulative percentage of mice with Racine score ≥ 4 was determined and compared among 3 groups. **D** Immunostaining of ZnT3 (green), counterstained with DAPI (blue) of the hippocampal dentate gyrus was performed on groups as indicated. ZnT3 expression was visualized at *iml*, *gcl*, and *hil*. Arrows indicate positive ZnT3 staining at *iml*. **E** Quantitative results of immunoreactivity of ZnT3 for iml (*left panel*) and gcl (*right panel*). Data presented as mean ± SEM.*p < 0.05, **p < 0.01, ***p < 0.001, ****p < 0.0001, n.s. not significant, using two-way ANOVA with *post-hoc* Fisher’s LSD test for Racine score and Unpaired T-test for each injection; one-way ANOVA, with Bonferroni correction as the *post-hoc* test for multiple comparisons for immunoreactivity of ZnT3. Abbreviations: iml, inner molecular layer; gcl, granule cell layer; hil, hilus

To test the anti-convulsant activity of J4 for focal seizures, a pentylenetetrazole (PTZ) kindling model of epilepsy that consists a schedule of an every-other-day, low-dose PTZ administration with a total of 21 injections was used on the wildtype (WT) mice. The effects of J4 treatment were compared to the vehicle-treated group (PTZ-Veh) and vigabatrin-treated group (PTZ-VGB). An experimental procedural diagram was shown in Fig. 1A. For each injection, the behavioral scoring of seizure of the mice was video-recorded, monitored and determined according to the Racine score [42]. Overall, the average seizure score of J4-treated group (PTZ-WT/J4) was significantly different from PTZ-WT/Veh group and PTZ-WT/VGB group (F(2, 330) = 33.36, *p* < 0.0001, two-way ANOVA). At injection 11, 16, and 17, PTZ-WT/J4 group showed significantly lower Racine score when compared to PTZ-WT/Veh and PTZ-WT/VGB (*p* = 0.023, *p* = 0.0088, and *p* = 0.0158 compared to PTZ-WT/Veh and *p* < 0.0001 compared to PTZ-WT/VGB, *post-hoc* Fisher’s LSD test). And at 8th, 12th, 13th, 14th, and 15th injections, PTZ-WT/J4 showed significantly lower Racine score compared to PTZ-WT/VGB (*p* < 0.05, two-way ANOVA with *post-hoc* Fisher’s LSD test) (Fig. 1B). Likewise, we found that PTZ-WT/J4 showed a significant reduction of the cumulative percentage of mice with Racine score ≥ 4 when compared to the PTZ-WT/Veh and PTZ-WT/VGB groups (F(2, 42) = 13.92, *p* < 0.0001; one-way ANOVA, *post-hoc* Bonferroni test, *p* = 0.0116 and *p* < 0.0001, respectively) (Fig. 1C).

Next, we used the zinc transporter 3 (ZnT3) expression to determine the mossy fiber sprouting (Fig. 1D). We found that after repetitive PTZ injections, increased ZnT3 immunoreactivity was shown in the dentate gyrus (DG) inner molecular layer (*iml*), as well as the granule cell layer (*gcl*) in the PTZ-WT/Veh group when compared to the Naïve-WT/Veh group (Fig. 1E). When compared to PTZ-WT/Veh group, PTZ-WT/VGB showed a slightly increased ZnT3 expression in the *iml*, whereas PTZ-WT/J4 group showed a trend of decreased ZnT3 expression. The results indicated that since both groups did not reach the statistical significance, suggesting VGB and J4 treatments cannot prevent the mossy fiber sprouting in the *iml* after 21 injections of PTZ. However, in the *gcl*, PTZ-WT/J4 group showed a significant decrease in ZnT3 immunoreactivity, suggesting mossy fiber sprouting was mitigated in this region of dentate gyrus.

### J4 decreased glial activation caused by PTZ-induced seizures

**Fig. 2:**
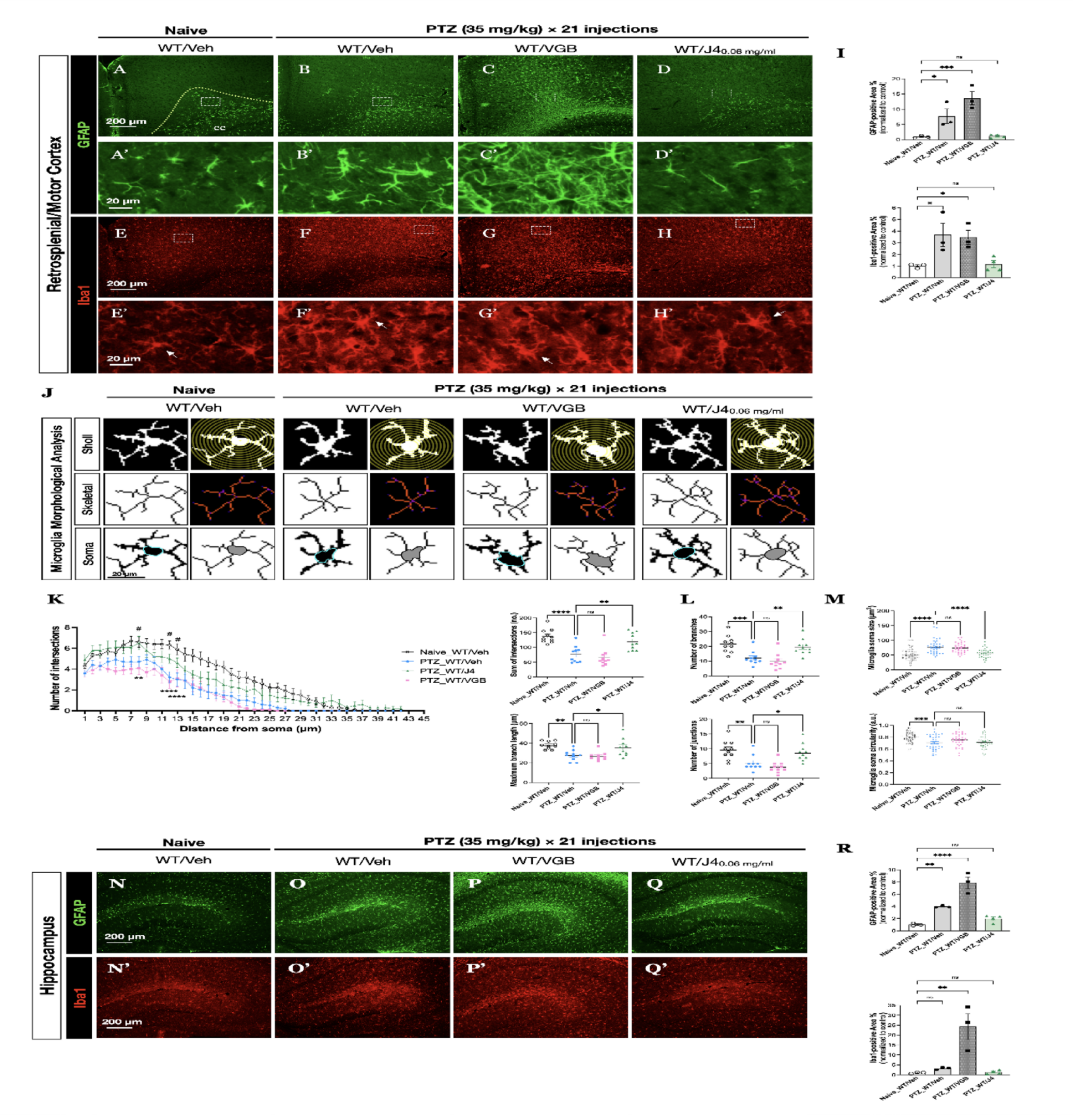
**A-D** Representative images of co-immunostaining of GFAP and (**E-H**) Iba1on brain sections from Naïve-WT/Veh (N = 3), PTZ-WT/Veh (N = 3), PTZ-WT/VGB (N = 3), and PTZ-WT/J4 (N = 4) at retrosplenial cortex are shown. **A’-D’** Magnified images of the area marked by the rectangle in A-D, respectively. **E’-H’** Magnified images of the area marked by the rectangle in E-H, respectively. Arrows indicate the isolated cells that underwent detailed analyses. **I** Quantitative analysis of percent coverage of GFAP staining *(upper panel*) and Iba1 staining (*lower panel*). **J** Representative single microglia cell underwent the Sholl analysis (*upper panel*), skeletal analysis (*middle panel*), and soma assessment (*lower panel*). **K** Quantitative results of Sholl analysis showing branching patterns of microglia in indicated groups. **p < 0.01, **** *p* < 0.0001 PTZ-WT/Veh v.s. Naïve-WT/Veh; # *p* < 0.05 PTZ-WT/J4 v.s. PTZ-WT/Veh, using two-way ANOVA, with Bonferroni correction as the *post-hoc* test for multiple comparisons. **L** Single cell skeletal analysis that quantifies microglial ramification, reflected by the number of branches (*upper panel*) and junctions (*lower panel*). **M** Soma body area and circularity were determined. **N-Q** Representative images of co-immunostained of brain sections for GFAP and (N’-Q’) Iba1 on hippocampal regions are shown. **R** Quantitative analyses of percent coverage of GFAP staining *(upper panel*) and Iba1 staining (*lower panel*) are shown. Data presented as ± SEM.*p < 0.05, **p < 0.01, ***p < 0.001, ****p < 0.0001, n.s. not significant, using one-way ANOVA, with Bonferroni correction as the *post-hoc* test for multiple comparisons. Abbreviations: CA1 sr, cornu ammonis 1stratum radiatum; DG mo, dentate gyrus molecular layer

In addition, in order to monitor the glial activation after PTZ-induced seizures, we performed the double labelling of fluorescent immunostaining of glial fibrillary acidic protein (GFAP) (Fig. 2A-D) and ionized calcium-binding adaptor molecule 1 (Iba1) of the cortical region (Fig. 2E-H) of each group. The results demonstrated that upon PTZ induction, PTZ-WT/Veh (Fig. 2B, F) and PTZ-WT/VGB (Fig. 2C, G) mice showed severe gliosis, while the PTZ-WT/J4 mice (Fig. 2D, H) exhibited a relatively reduced GFAP- and Iba1-positive staining in the cortical region near the white matter region. The quantitative results validated that after PTZ induction, the PTZ-WT/Veh group showed a significantly increased GFAP-positive area percentage (F(3, 9) = 16.48, *p* = 0.0005; one-way ANOVA, *post-hoc* Bonferroni test, *p* = 0.0373) (Fig. 2I, *upper panel*), as well as Iba1-positive area percentage (F(3, 9) = 6.44, *p* = 0.0128; one-way ANOVA, *post-hoc* Bonferroni test, *p* = 0.0314), when compared to Naïve-WT/Veh group (Fig. 2I, *lower panel*). In contrast, PTZ-WT/J4 group showed no significant changes in glia immunoreactivity when compared to Naïve-WT/Veh group.

Furthermore, we assessed the morphology of microglia, using Sholl, skeleton analyses and quantified the soma body size and circularity (Fig. 2J). The Sholl analysis, which generates concentric circles around the cell body and determines the number of intersections, showed the extent of branching that each microglia has (Fig. 2J, *upper panel*; Fig. 2K). PTZ-WT/Veh and PTZ-WT/VGB groups exhibited significantly different branching pattern, when compared to the Naïve-WT/Veh (F(3, 1512) = 153.2, *p* < 0.0001; two-way ANOVA). In particular, at distances of 8 μm, 12 μm and 13 μm away from the soma, PTZ-WT/Veh showed a prominent reduced number of intersections, when compared to Naïve-WT/Veh (F(2, 1134) = 122.2, *p* < 0.0001, one-way ANOVA, *post-hoc* Bonferroni test, *p* = 0.0061, *p* < 0.0001, and *p* < 0.0001, respectively); meanwhile PTZ-WT/J4 significantly increased the number of intersections at these 3 locations, when compared to PTZ-WT/Veh (F(2, 1134) = 122.2, *p* < 0.0001, one-way ANOVA, *post-hoc* Bonferroni test, *p* = 0.0103, *p* = 0.0103, and *p* = 0.0278, respectively).

Single cell skeletal analysis was then used to quantify microglial ramification, which were reflected by the number of branches and junctions (Fig. 2J, *middle panel*; Fig. 2L). PTZ-WT/J4 showed an increased number of branches and junctions, when compared to PTZ-WT/Veh (F(3, 36) = 11.88, *p* < 0.0001, *post-hoc* Bonferroni test, *p* = 0.0086 ; and F(3, 36) = 10.66, *p* < 0.0001, one-way ANOVA, *post-hoc* Bonferroni test, *p* = 0.0155). Lastly, microglia soma body area and circularity were determined (Fig. 2J, *lower panel*; Fig. 2M). PTZ-WT/J4 showed a decreased microglial cell body size when compared to PTZ-WT/Veh group (F(3, 156) = 19.10, *p* < 0.0001, one-way ANOVA, *post-hoc* Bonferroni test, *p* < 0.0001).

Likewise, in the hippocampal regions, when compared to the Naïve-WT/Veh group (Fig. 2N) the PTZ-WT/Veh group (Fig. 2O) and the PTZ-WT/VGB group (Fig. 2P) showed significantly higher GFAP-staining area percentage, while PTZ-WT/J4 group (Fig. 2Q) showed equal amount of glial activation as Naïve-WT/Veh group F(3, 9) = 38.26, *p* < 0.0001, one-way ANOVA, *post-hoc* Bonferroni test, *p* = 0.0068, *p* < 0.0001, and *p* = 0.5118, respectively) (Fig. 2R, *upper panel*). On the other hand, no difference in Iba1-staining area percentage was observed in PTZ-WT/Veh and PTZ-WT/J4 groups (Fig. 2O**’** and Fig. 2R, *lower panel*). We also noticed that the PTZ-WT/VGB group has the most prominent activation of astroglia and microglia F(3, 9) = 13.70, *p* < 0.0011, one-way ANOVA, *post-hoc* Bonferroni test, *p* = 0.0014 (Fig. 2P’ and Fig. 2R, *lower panel*), which may correspond to the seizure severity observed in Fig. 1B, C.

### Tsc2^+/–^ mice was more susceptible to seizure induction by a sub-convulsive dose of PTZ

**Fig. 3:**
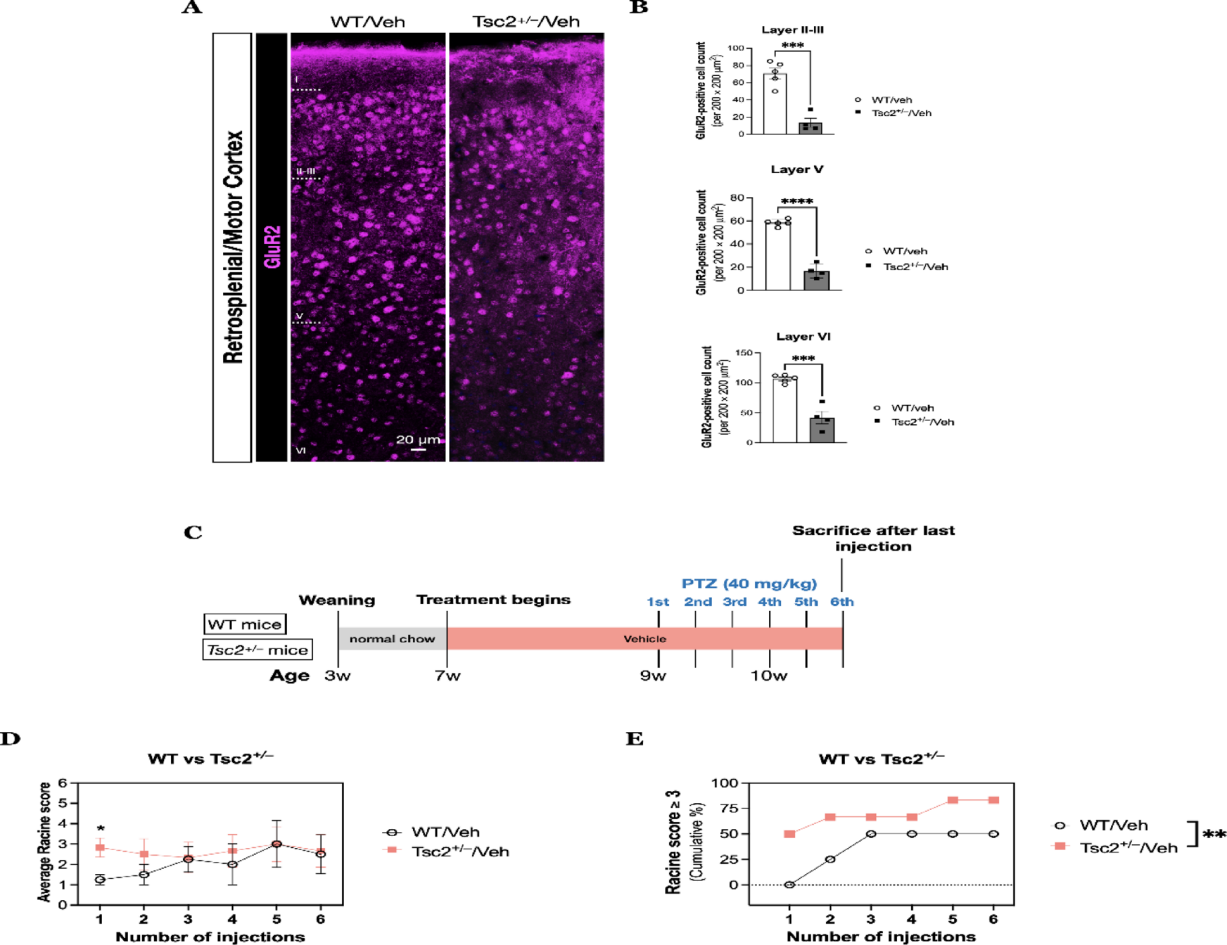
**A** Fluorescent immunohistochemistry on coronal sections of WT (*N* = 5) and *Tsc2*^+/–^ mice (*N* = 4) for GluR2 was performed. **B** Quantitative results of GluR2-positive cells for cortical layer II-III (*top panel*), layer V (*middle panel*), and layer VI (*bottom panel*) were determined for each group. **C** The experimental procedural diagram for determining the differences in seizure susceptibility between WT and *Tsc2*^+/–^ mice. Seizure behavior was determined in WT (*N* = 4) and *Tsc2*^+/–^ mice (*N* = 6) with the vehicle treatment after six intraperitoneal injection of pentylenetetrazol (PTZ) with a dose of 40 mg/kg. **D** The average Racine score for WT and *Tsc2*^+/–^ mice for each injection. **E** The cumulative percentage of animals that exhibited Racine score ≥ 3 during the PTZ induction paradigm. Data presented as mean ± SEM.*p < 0.05, ** *p* < 0.01, ***p < 0.001, ****p < 0.0001, n.s. not significant, using Unpaired T-test and two-way ANOVA

We next want to test whether J4 has similar anti-convulsant and/or anti-inflammatory effects on epilepsies with a genetic cause. The *Tsc2*^+/–^ mouse model has been shown to have no spontaneous seizures [46, 47], but a study has demonstrated that *Tsc2*^+/–^ rats have a lower seizure threshold after PTZ chemical kindling [48]. In addition, both patients and rodents of TSC have been reported to exhibit increased network excitability [49–54]. Since defects in glutamate receptors may contribute to enhanced excitation, we next explored the alterations in GluR2 protein expression of *Tsc2*^+/–^ mice before and after PTZ injections. GluR2 is a subunit for α-amino-3-hydroxy-5-methyl-4-isoxazoleproprionic acid receptor (AMPARs), and its absence or low expression in AMPARs renders Ca^2+^ permeability into post-synaptic neurons, mediating excitatory neurotransmission [55]. We confirmed that *Tsc2*^+/–^ mice in this study indeed showed a decreased GluR2 expression (Fig. 3A, B), which may render a low seizure threshold when exposed to a chemical assault such as PTZ. We first tested the different doses of PTZ administration to determine the vulnerability of *Tsc2*^+/–^ mice to PTZ. We found that a single intraperitoneal injection of PTZ at high doses (50, 60, 70 and 80 mg/kg) would induce an acute, severe seizure behavior in both WT and *Tsc2*^+/–^ mice, regardless of the age of the mice. All mice reached Racine score ≥ 4, so we recorded the latency to first minimal clonic seizure (MCS) and the total time duration of seizures for each animal. When compared to WT mice, *Tsc2*^+/–^ mice did not show significant differences at 4-week-old in the latency to first MCS (Supplemental Fig. 1A, upper panel) and the total duration time of seizures (Supplemental Fig. 1 A, lower panel). Likewise, the two groups did not differ at the age of 8 weeks as well (Supplemental Fig.1 B). Therefore, a sub-convulsive dose of PTZ (40 mg/kg) was used to differentiate the response to PTZ between WT and *Tsc2*^+/–^ mice.

We used a sub-convulsive dose of PTZ-induced kindling protocol to observe the seizure score for each group (Fig. 3C). PTZ was injected every other day for 6 times at a sub-convulsive dose (40 mg/kg, i.p.), which WT/Veh was expected to exhibit no convulsive response or signs of myoclonic jerks (stage 1). As expected, the WT/Veh mice showed no obvious response during the first and second injections of PTZ, while *Tsc2*^+/–^/Veh mice showed increased Racine score (Fig. 3D). At the first injection of PTZ, the seizure behavior of *Tsc2*^+/–^/Veh mice had an average Racine score of 2.83 ± 0.48, while that of WT/Veh group was 1.25 ± 0.25 (*p* = 0.036, unpaired T-test). We found that the percentage of animals showed notable seizure behavior (Racine score ≥ 3) was higher in *Tsc2*^+/–^ mice during the whole PTZ administration paradigm (F(1, 5) = 34.40, *p* = 0.002, two-way ANOVA) (Fig. 3E).

### J4 ameliorated seizure severity in the Tsc2^+/–^ mice evoked by PTZ injections

**Fig. 4:**
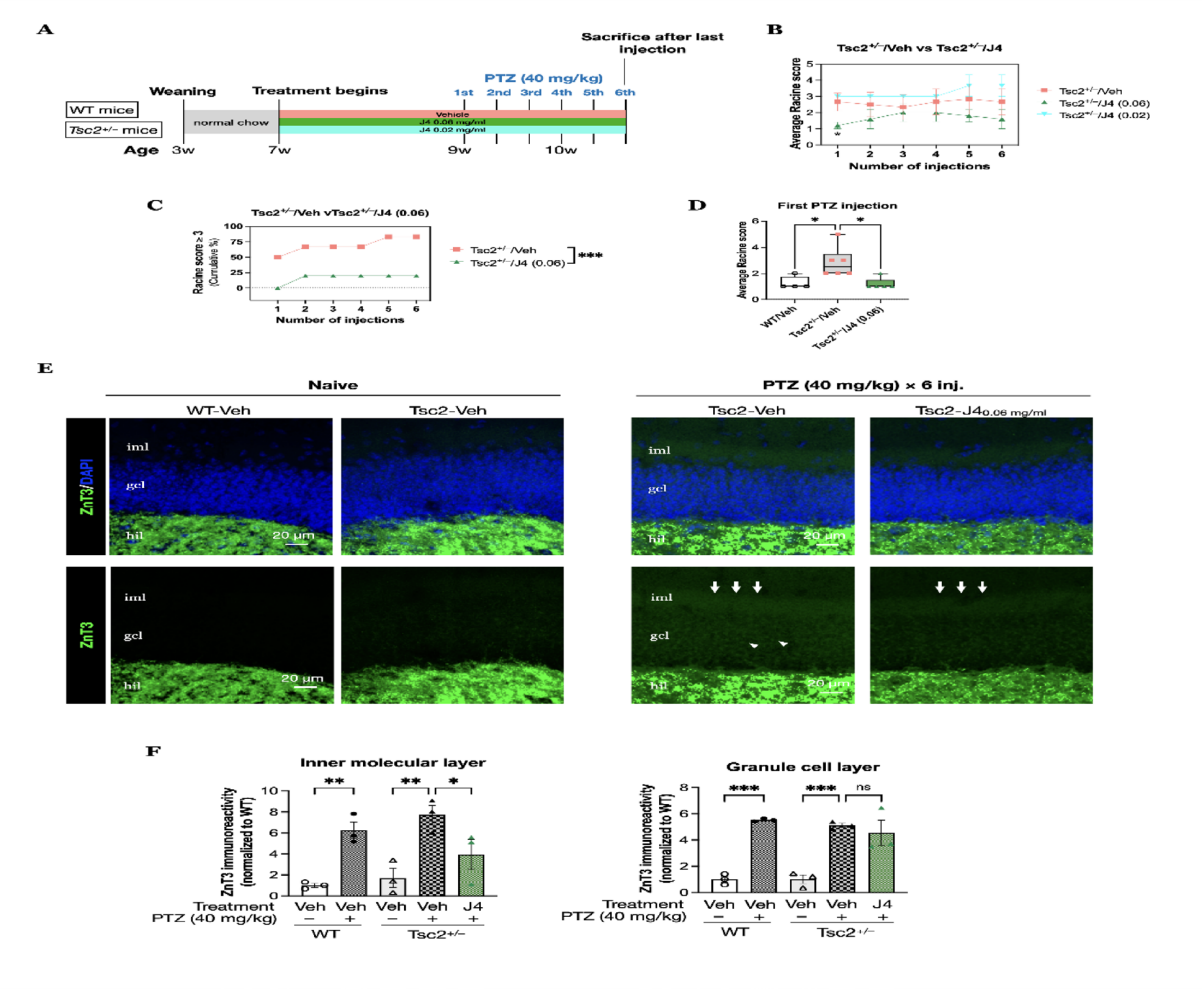
**A** The experimental procedural diagram for determining the seizure susceptibility of *Tsc2*^+/–^ mice with J4 treatments. Seizure behavior was determined in PTZ-Tsc2^+/–^/Veh (*N* = 6), PTZ-Tsc2^+/–^/J4 (0.02 mg/ml) (*N* = 3) and PTZ-Tsc2^+/–^/J4 (0.06 mg/ml) (*N* = 5) after six intraperitoneal injection of pentylenetetrazol (PTZ) with a dose of 40 mg/kg. **B** The average Racine score for *Tsc2*^+/–^ mice with 3 different treatments is shown for each injection. **C** The cumulative percentage of animals displayed a Racine score ≥ 3 is shown for every injection for the Tsc2^+/–^/Veh and Tsc2^+/–^/J4 (0.06). **D** Comparison of the 3 groups for their average PTZ-induced seizure score at the first injection. **E** Immunostaining of ZnT3 (green), counterstained with DAPI (blue) of the hippocampal dentate gyrus was performed on groups as indicated. ZnT3 expression was visualized only in *hil* in Naïve groups (*left panel*), while it was visualized in *iml* (arrows) and in *gcl* (arrowheads) after 6 injections of 40 mg/kg PTZ (*right panel*). **F** Quantitative results of immunoreactivity of ZnT3 are shown. Data presented as mean ± SEM, *p < 0.05, using two-tailed unpaired T-test to compare PTZ-Tsc2^+/–^/Veh and PTZ-Tsc2^+/–^/J4 (0.06 mg/ml) for each injection; and using two-way ANOVA, with Bonferroni correction as the *post-hoc* test for multiple comparisons; and Kruskal-Wallis test with *post-hoc* Dunn’s test for comparisons of 3 groups. *p < 0.05, **p < 0.01, ***p < 0.001, n.s. not significant, using one-way ANOVA, with Bonferroni correction as the *post-hoc* test for multiple comparisons. Abbreviations: iml, dentate gyrus inner molecular layer; gcl, granule cell layer; hil, hilus

Next, we aimed to explore the potential of J4 in reducing the susceptibility to seizure triggers in *Tsc2*^+/–^ mice under the induction by PTZ. In this part of experiment, we used two doses of J4, 0.06 mg/ml and 0.02 mg/ml and used 6 PTZ injections with a sub-convulsive dose, 40 mg/kg (Fig. 4A). The groupings are as follows: WT mice with no PTZ injections (Naïve-WT/Veh), WT mice with 6 PTZ injections (PTZ-WT/Veh), *Tsc2*^+/–^ mice with no PTZ injections (Naïve-Tsc2^+/–^/Veh), *Tsc2*^+/–^ mice after 6 PTZ injections (PTZ-Tsc2^+/–^/Veh), *Tsc2*^*+/–*^ mice pre-treated with 0.02 mg/ml of J4 and with 6 PTZ injections (PTZ-Tsc2^+/–^/J4 (0.02)), and *Tsc2*^+/–^ mice pre-treated with 0.06 mg/ml of J4 and with 6 PTZ injections (PTZ-*Tsc2*^*+/–*^/J4 (0.06)). When compared to the PTZ-Tsc2^+/–^/Veh mice, PTZ-Tsc2^+/–^/J4 (0.06) mice showed a milder seizure behavior, whereas PTZ-Tsc2^+/–^/J4 (0.02) did not show differences, indicating the treatment of J4 in the dose of 0.06 mg/ml was more effective in protecting *Tsc2*^+/–^ mice from PTZ-triggered convulsive behavior (Fig. 4B) (*p* = 0.017, unpaired T test). Altogether, PTZ-Tsc2^+/–^/Veh mice showed a significantly increased percentage of animals with Racine score ≥ 3 when compared to the PTZ-WT/Veh mice (F(2, 10) = 56.53, *p* = 0.0002, two-way ANOVA, *post-hoc* Bonferroni test), and PTZ-Tsc2^+/–^/J4 (0.06) mice showed a significantly decreased percentage of animals with Racine score ≥ 3 when compared to PTZ-Tsc2^+/–^/Veh mice (F(2, 10) = 56.53, *p* < 0.0001, two-way ANOVA, *post-hoc* Bonferroni test) (Fig. 4C). At the first injection, the average Racine score for PTZ-Tsc2^+/–^/Veh mice was significantly higher than PTZ-WT/Veh and PTZ-Tsc2^+/–^/J4 (0.06) mice (*p* < 0.05, Kruskal-Wallis test, *post-hoc* Dunn’s test) (Fig. 4D). We also used ZnT3 to observe the mossy fiber sprouting at DG (Fig. 4E). We found that the inner molecular layer of DG showed a significantly increased ZnT3 immunoreactivity in both PTZ-WT/Veh and PTZ-Tsc2^+/–^/Veh, when compared to the Naïve animals; and a significant reduction of ZnT3 expression in PTZ-Tsc2^+/–^/J4 (0.06) group (F(4, 10) = 9.55, *p* = 0.0019, one-way ANOVA, *post-hoc* Bonferroni test, *p =* 0.0497) (Fig. 4F, *left panel*). On the other hand, in the granule cell layer, the J4-treated group did not significantly differ from the Veh-treated group after PTZ-induced seizures (Fig. 4F, *right panel*).

### J4 decreased the number of degenerating cells and prevented cortical cell loss resulted from PTZ-induced seizures

**Fig. 5:**
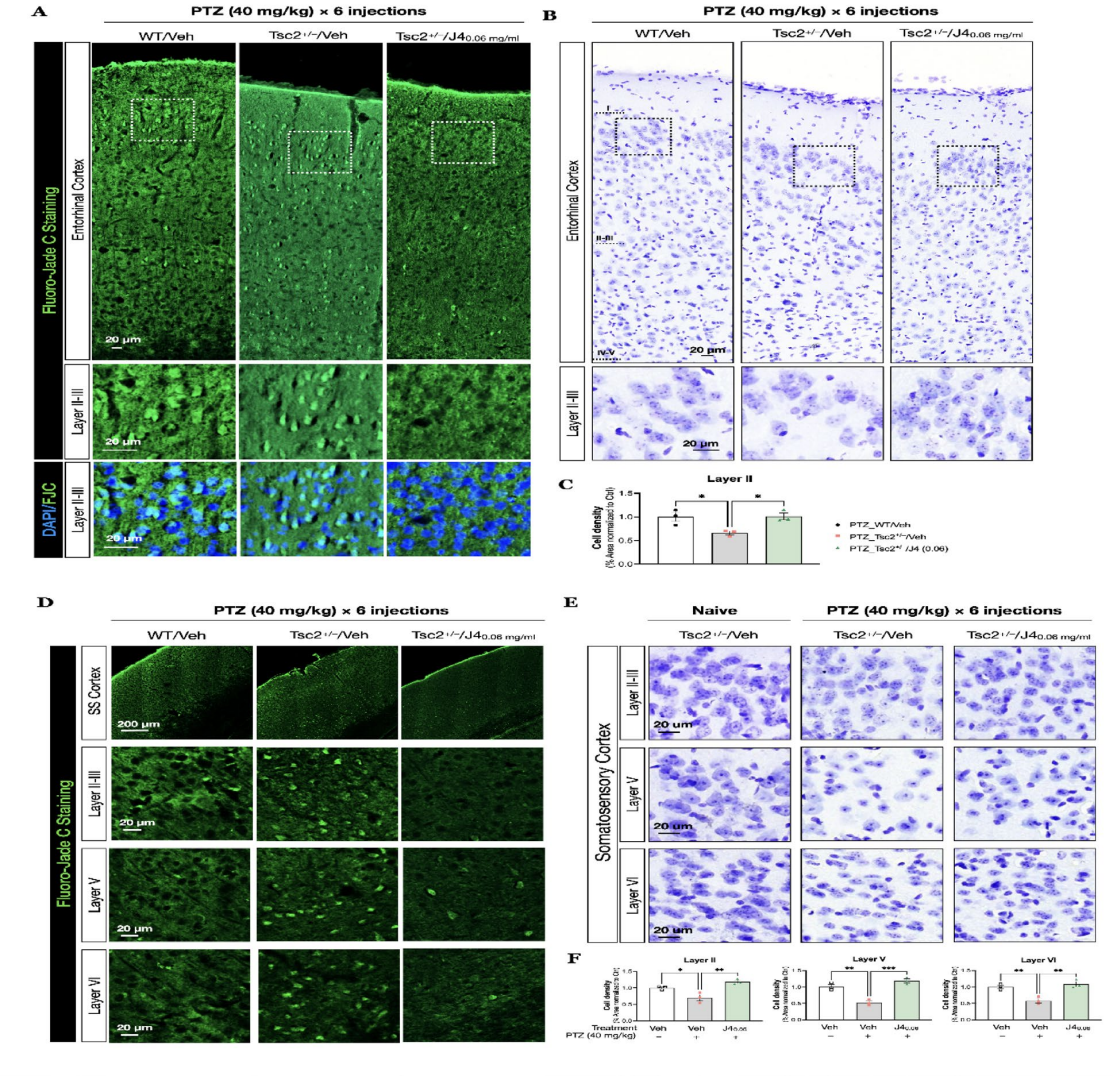
**A** FJC staining (green), counterstained with DAPI (blue) was performed to analyze the degenerating cells at the entorhinal cortex of the indicated groups. Layer II-III, marked by the rectangle, was magnified and shown in the lower panels. **B** Nissl-staining was performed to assess the neuronal loss at the corresponding region. **C** The quantitative analysis of cell density at the cortical layer II-III is shown for PTZ-WT/Veh (*N* = 3), PTZ-Tsc2^+/–^/Veh (*N* = 3), PTZ- Tsc2^+/–^/J4 (0.06) (*N* = 4). **D** FJC staining (green), counterstained with DAPI (blue) was performed at the somatosensory cortex of the indicated groups. **E** Nissl-staining was performed to compare these groups Naive-Tsc2^+/–^/Veh (*N* = 3), PTZ-Tsc2^+/–^/Veh (*N* = 3), PTZ- Tsc2^+/–^/J4 (0.06) (*N* = 4). **F** Quantitative results of cell density in groups as indicated are shown. Data presented as mean ± SEM, *p < 0.05, ** *p* < 0.01, ***p < 0.001, ****p < 0.0001, n.s. not significant, using one-way ANOVA, with Bonferroni correction as the *post-hoc* test for multiple comparisons. Abbreviations: FJC, Fluoro-Jade staining

Neurodegeneration with the hippocampus and entorhinal cortex is a hallmark of chronic temporal lobe epilepsy (TLE) [56]. Therefore, we then performed Fluoro-Jade C (FJC) staining and analyzed the degenerating cells at the entorhinal cortex (Supplementary Fig. 2A-C). When compared to the Naïve-WT/Veh (Supplementary Fig. 2 A), PTZ-WT/Veh showed increased FJC-positive cells at layer II-III of the entorhinal cortex (Supplementary Fig. 2 B, Fig. 5A, *left panel*). After pre-treatment of J4, the number of FJC-positive cells decreased (Supplemental Fig. 2 C). Whereas in *Tsc2*^+/–^ mice, we compared the following groups: PTZ-Tsc2^+/–^/Veh and PTZ-Tsc2^+/–^/J4 (0.06). After PTZ injections, FJC-positive cells notably increased in PTZ-Tsc2^+/–^/Veh, when compared to PTZ-WT/Veh (Fig. 5A, *middle panel*). This indicated that compared to WT mice, *Tsc2*^+/–^ mice showed a more aggravated cell degeneration after PTZ-induced seizures. Pretreatment of J4 in *Tsc2*^*+/–*^ mice was able to alleviate the cell degeneration (Fig. 5A, *right panel*).

It has been known that PTZ-induced seizures cause neuronal cell loss [57, 58], and to confirm the cell loss occurs due to neurodegeneration caused by seizures, we assessed the cell density at the entorhinal cortical region (Fig. 5B). We compared the number of Nissl-positive staining cells for the following groups: Naïve-Tsc2^+/–^/Veh, PTZ-Tsc2^+/–^/Veh, PTZ-Tsc2^+/–^/J4 (0.02)), and PTZ-Tsc2^+/–^/J4 (0.06) at the entorhinal cortex. We assessed the cortical layers II-III and layer IV-V (Fig. 5B). We found that PTZ-Tsc2^+/–^/Veh group showed a significant decrease in the cell density when compared to the Naïve-Tsc2^+/–^/Veh at layer II (Fig. 5B, *middle panel*). The pretreatment of J4 with the dose of 0.02 mg/ml did not have effects on the cell density (data not shown), whereas J4 treatment with the dose of 0.06 mg/ml significantly increased the cell number in the cortical layer II of *Tsc2*^+/–^ mice after PTZ-induced seizures (F(2, 6) = 7.926, *p* = 0.0207, one-way ANOVA, *post-hoc* Bonferroni test, *p* = 0.0296 and *p* = 0.0253). (Fig. 5B, *right panel*; Fig. 5C)

Similarly, we found increased FJC-positive cells at the somatosensory cortex of PTZ-Tsc2^+/–^/Veh mice, when compared to PTZ-WT/Veh (Fig. 5D, *left and middle panels*). PTZ-Tsc2^+/–^/J4 (0.06) showed reduced FJC-positive cells in layers II-VI (Fig. 5D, *right panel*). From Nissl staining result, we also confirmed that the cell loss was significantly attenuated in the J4-treated group (Fig. 5E) at layer II (F(3, 8) = 15.54, *p* = 0.004, one-way ANOVA *post-hoc* Bonferroni test, *p* = 0.003) (Fig. 5F, *left panel*); layer V (F(3, 8) = 33.26, *p* = 0.0006, one-way ANOVA *post-hoc* Bonferroni test, *p* = 0.0004) (Fig. 5F, *middle panel*); and layer VI (F(3, 8) = 17.17, *p* = 0.0033, one-way ANOVA, *post-hoc* Bonferroni test, *p* = 0.003) (Fig. 5F, *right panel*). However, we did not observe prominent differences at the hippocampal regions (Supplementary Fig. 3).

### Increased GluR2 and decreased DCX expression were observed in J4-treated Tsc2^+/–^ mice following PTZ-induced seizures

**Fig. 6:**
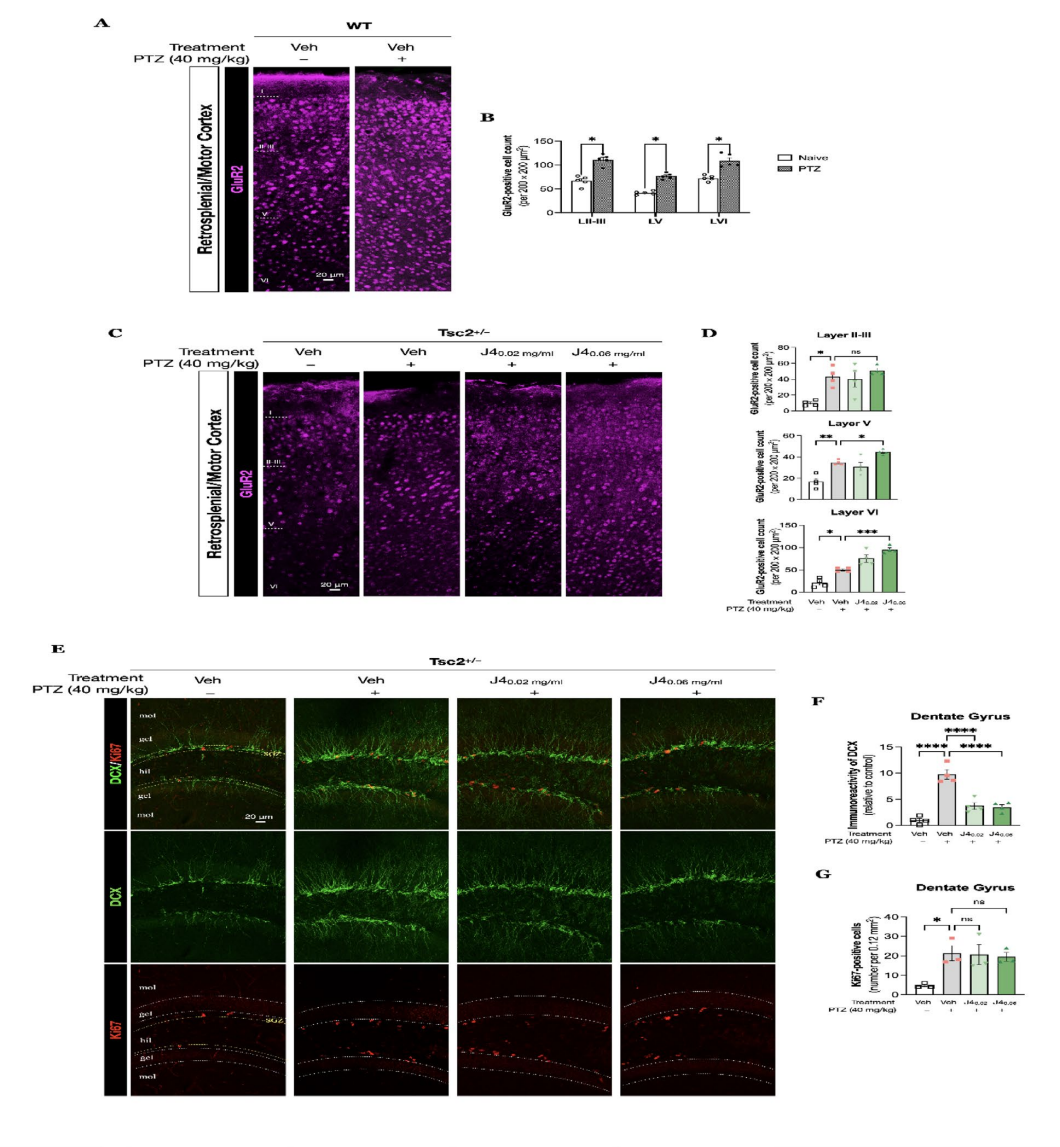
**A** Fluorescent immunohistochemistry on coronal sections of WT mice (*N* = 5) for GluR2 was performed to visualize the GluR2-positive cells in response to the PTZ injection. **B** Quantitative analysis was performed to determine GluR2-positive cells in cortical layers (II-III, V, and VI). **C** Fluorescent immunohistochemistry of *Tsc2*^+/–^ mice with different treatments (Vehicle, J4 (0.02 mg/ml), and J4 (0.06 mg/ml) for GluR2 was performed to visualize the GluR2-positive cells for each group (*N* = 4) in response to the PTZ injection. **D** Quantitative analysis was performed to determine GluR2-positive cells in cortical layers (II-III, V, and VI) for each group. **E** Fluorescent immunohistochemistry of DCX (green), co-labeled with Ki67 (red), of the hippocampal dentate gyrus of each group was performed. Quantitative results of immunoreactivity of DCX **F** and Ki67-positive cells **G** are shown. Data presented as mean ± SEM, *p < 0.05, ** *p* < 0.01, ***p < 0.001, ****p < 0.0001, n.s. not significant using unpaired T test and one-way ANOVA, with Bonferroni correction as the *post-hoc* test for multiple comparisons. Abbreviations: DCX, doublecortin

Next, we probed into the possible mechanisms of J4 in *Tsc2*^*+/–*^ mice. Since the expression of GluR2-containing-Ca^2+^-impermeable AMPARs are considered to be important for synaptic transmission and prevent cell loss due to excitotoxicity in mature brains [55, 59], we first determined the level of GluR2 expression in WT mice after PTZ-induced seizures (Fig. 6A). As the results showed, the GluR2-positive cells significantly increased at cortical layers II-III, V and VI after PTZ injections (*p* < 0.05, unpaired T test), suggesting that the glutamatergic neurons in WT mice counteracted the hyperexcitability by helping reducing the calcium permeability of AMPARs, thereby protecting the neurons from excitotoxic damage (Fig. 6B). Similarly, in *Tsc2*^+/–^ mice, the GluR2 expression was also increased after PTZ injections at cortical layers II-III, V and VI (Fig. 6C), and PTZ-Tsc2^+/–^/J4 (0.06) mice showed significantly increased GluR2 expression at cortical layers V (F(3, 12) = 25.09, *p* < 0.0001, one-way ANOVA, *post-hoc* Bonferroni test, *p* < 0.029), and VI (F(3, 12) = 35.75, *p* < 0.0001, one-way ANOVA, *post-hoc* Bonferroni test, *p* = 0.0004), when compared to the PTZ-Tsc2^+/–^/Veh mice (Fig. 6D). In contrast, PTZ-Tsc2^+/–^/J4 (0.02) mice did not show differences when compared to the PTZ-Tsc2^+/–^/Veh mice.

Moreover, we performed double labelling of Ki67 doublecortin (DCX) to monitor J4 treatment’s effect on seizure-induced neurogenesis (Fig. 6E). We first explored the DCX immunoreactivity after PTZ injections and compared to Naïve-Tsc2^+/–^/Veh, and found that PTZ- Tsc2^+/–^/Veh group exhibited tremendously increased immunoreactivity of DCX (F(3, 12) = 34.14, *p* < 0.0001, one-way ANOVA, *post-hoc* Bonferroni test, *p* < 0.0001). Then, DCX expression of J4-treated groups was compared to that of the PTZ- Tsc2^+/–^/Veh group. We found that J4-treated groups exhibited a notable decreased DCX expression after PTZ-induced seizures F(3, 12) = 34.14, *p* < 0.0001, one-way ANOVA, *post-hoc* Bonferroni test, *p* < 0.0001 and *p* < 0.0001) (Fig. 6F). Meanwhile, the number of Ki67-positive cells remained unchanged after J4 pretreatment (Fig. 6G).

### J4 mitigated severe gliosis in Tsc2^+/–^ mice after PTZ-induced seizures

**Fig. 7:**
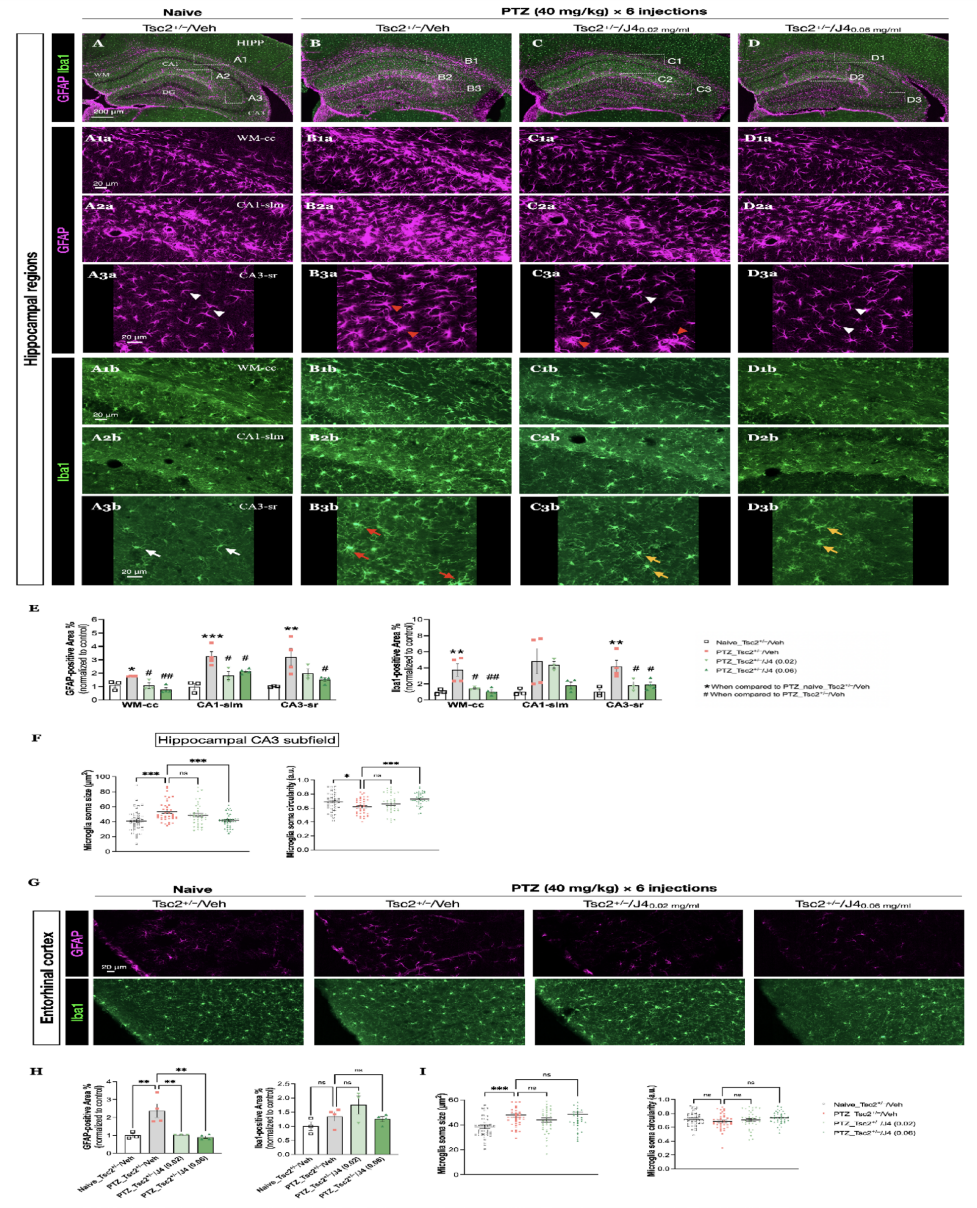
**A-D** Co-immunostaining of GFAP (magenta) (A1a-D3a) and Iba1 (green) (A1b-D3b) of the hippocampal region of the indicated groups was performed, and 3 magnified images from the marked regions of each group are shown (A1-A3, B1-B3, C1-C3, D1-D3). Rectangle 1, 2, and 3 indicate the WM-cc, CA1-slm, and CA3-sr regions, respectively. Visualization of GFAP-positive cells shows non-reactive astrocytes, indicated by the white arrowheads (A3a, C3a, D3a) and reactive astrocytes, indicated by the red arrowheads (B3a, C3a). Visualization of Iba1-positive cells shows resting microglia, indicated by the white arrows (A3b); and activated microglia with high ramification, indicated by the red arrows (B3b), and activated microglia with less ramification, indicated by the yellow arrows (C3b, D3b). **E** Quantitative results of GFAP- (*left panel*) and Iba1-positive area percentage (*right panel*) of each brain region are shown. **F** Quantitative results of microglia soma size (*left panel*) and circularity (*right panel*) at CA3 subfield are shown. Data presented as mean ± SEM, *p < 0.05, ** *p* < 0.01, ***p < 0.001, ****p < 0.0001, n.s. not significant, using one-way ANOVA, with Bonferroni correction as the *post-hoc* test for multiple comparisons

Seizures are also known to cause astrogliosis and microgliosis in the hippocampal regions [60, 61]. Therefore, we used antibodies GFAP and Iba1 to monitor the pathological changes in astrocytes and microglia, respectively, in *Tsc2*^*+/–*^ mice before and after PTZ injections, either with or without pretreatment (Fig. 7A-D). We assessed corpus callosum (WM-cc) (Fig. 7 A1) and two hippocampal regions, cornu ammonis 1 stratum lacunosum moleculare (CA1-slm) (Fig. 7A2), and cornu ammonis 3 stratum radiatum (CA3-sr) (Fig. 7 A3). When compared to Naïve-Tsc2^+/–^/Veh group (Fig. 7 A1a, A2a, A3a), the PTZ-Tsc2^+/–^/Veh group showed a prominent astroglial activation at the WM-cc and CA1-slm and a slight astroglial activation at the CA3-sr shown by the GFAP-positive staining (Fig. 7 B1a, B2a, B3a). With the pretreatment of J4 at the dose of 0.02 mg/ml (PTZ-Tsc2^+/–^/J4 (0.02)), the mice showed a decreased GFAP-positive staining area (Fig. 7 C1a C2a, C3a). We also evaluated the effect of J4 at the dose of 0.06 mg/ml (PTZ-Tsc2^+/–^/J4 (0.06)) and found that this dose can lower the astoglial activation with a better efficacy at CA3-sr (Fig. 7 D1a, D2a, D3a).

Moreover, we also explored whether microglia are affected after PTZ-induced seizures in *Tsc2*^+/–^ mice and whether the J4 treatment has an effect on these glial cells. Similarly, PTZ-Tsc2^+/–^/Veh group showed an increased Iba1-positive area percentage and ramified microglia at the WM-cc, CA1-slm, and CA3-sr when compared to the Naïve-Tsc2^+/–^/Veh group (Fig. 7A1b, A2b, A3b, B1b, B2b, B3b). When pre-treated with J4, at the dose of 0.02 mg/ml, the mice showed a significant reduction in Iba1 immunoreactivity at WM-cc and CA3-sr, but not CA1-slm ( Fig. 7C1b, C2b, C3b). On the other hand, at the dose of 0.06 mg/ml, PTZ-Tsc2^+/–^/J4 (0.06) group showed similar Iba1 immunoreactivity and microglia morphology as the Naïve-Tsc2^+/–^ Veh mice (Fig. 7 D1b, D2b, D3b). We further confirmed the results after quantitative analyses of GFAP- and Iba1-positive area percentage (Fig. 7E).

We further analyzed microglia soma size and circularity to determine the microglial activation. We found that the soma in PTZ-Tsc2^+/–^/Veh group were enlarged and J4 (0.06 mg/ml) pretreatment significantly reduced the soma size, but not J4 (0.02 mg/ml) pretreatment (F(3, 156) = 7.968, *p* < 0.0001, one-way ANOVA, *post-hoc* Bonferroni test, *p* = 0.0002, *p* = 0.0003, and *p* = 0.3666, respectively) (Fig. 7F, *left panel*). Likewise, PTZ-induction caused the microglia soma circularity to decrease in *Tsc2*^+/–^ mice and pretreatment of J4 (0.06 mg/ml) alleviated the abnormality of soma shape, but not J4 (0.02 mg/ml) pretreatment (F(3, 156) = 6.383, *p* = 0.0004, one-way ANOVA, *post-hoc* Bonferroni test, *p* = 0.0198, *p* = 0.0001, and *p* = 0.3657, respectively) (Fig. 7F, *right panel*).

We also assessed the glial activation at the entorhinal cortex and found similar results as the hippocampal regions (Fig. 7G). PTZ-Tsc2^+/–^/Veh mice exhibited increased GFAP-positive area percentage and J4 pretreatments, either 0.02 mg/ml or 0.06 mg/ml, reduced the GFAP-positive staining F(3, 10) = 10.17, *p* = 0.0022, one-way ANOVA, *post-hoc* Bonferroni test, *p* = 0.0057, *p* = 0.0064, and *p* = 0.0021, respectively) (Fig. 7H, *left panel*). However, we did not observe changes in Iba1-positive area percentage in all 4 groups (Fig. 7H, *right panel*), though the soma size of microglia was significantly increased in PTZ-Tsc2^+/–^/Veh mice (Fig. 7I, *left panel*), and the soma circularity stayed unchanged (Fig. 7I, *right panel*). These results indicate that microglial activation in the cortical region was not as prominent as in the hippocampal regions after PTZ induction.

## Discussion

This study investigated the anti-convulsant potential of a novel compound, J4, which acts through inhibiting the ENT1, in two types of seizure/epilepsy models. Previously, J4 has been shown to have anti-convulsant effects in both the acute PTZ-induced seizure model and the PTZ-induced kindling model [40]. In line with these results, this study also demonstrated that J4 was able to ameliorate the seizure severity in a chronic epileptic model, which was induced by a repetitive low dose of PTZ injections. In the PTZ kindling model (21 injections) in our study, we observed ZnT3-positive puncta were significantly decreased in the *gcl* of DG following treatment, while no significant reduction in mossy fiber sprouting in the *iml* of DG after J4 treatment. One possible explanation is that while J4 may modulate synaptic zinc release in dentate granule cell terminals or granule cell excitability, it does not substantially alter structural remodeling of mossy fibers. This selective effect suggests that J4 may exert a disease-modifying effect rather than fully anti-epileptogenic role in this model.

More importantly, this study also probed into the effects of J4 in a mouse model of TSC, which represents the most common genetic cause for pediatric epilepsy, and compared the effects of J4 treatment with the VGB treatment, which is the recommended first-line anticonvulsant therapy for infantile spasms in TSC. In the present study, we found that J4 pretreatment in *Tsc2*^+/–^ mice lowered the seizure severity upon PTZ insults and ameliorated mossy fiber reorganization at iml of DG (Fig. 4). In addition, J4 pretreatment prevented cortical cell loss due to neurodegeneration caused by PTZ-induced seizures in *Tsc2*^+/–^ mice (Fig. 5). We analyzed the entorhinal, somatosensory cortices and hippocampal regions, including CA1, CA3, and DG. While cortical neuronal cell loss was observed in cortical areas, Nissl staining of the hippocampal subfield areas did not reveal detectable differences compared with controls, likely because 6 PTZ injections were insufficient to induce hippocampal neuronal loss.

TSC patients and other mouse models have been shown to exhibit neuronal hyperexcitability because of the impairment in excitation and inhibition balance (E/I balance) [62–64]. In this present study, we further validated that *Tsc2*^+/–^ mice are intrinsically hyperexcitable since the GluR2 expression is differentially expressed compared to the WT mice. A higher expression of GluR2-lacking AMPARs has been shown in the immature brain compared with the adult brain, indicating a higher susceptibility to seizures in the neonatal brain due to the hyperexcitability [65]. Therefore, we suppose that the GluR2 subunit maturation is deficient in *Tsc2*^+/–^ mice during the developmental stages, and in turn may contribute to the hypersensitivity of seizure triggers. Hence, at the first PTZ injection, *Tsc2*^+/–^ mice showed a higher percentage of animals with more severe seizure behavior than WT mice (Fig. 3A-E). Our results also demonstrated that in response to PTZ, WT mice exhibited a higher level of GluR2 expression than *Tsc2*^+/–^ mice, which may represent a compensatory protective response to limit calcium influx; and that *Tsc2*^+/–^ mice showed a deficient function in responding to PTZ insults. Pretreatment of J4 helped *Tsc2*^+/–^ mice to enhance GluR2 expression, and prevented excitotoxicity (Fig. 6A-C). The mechanisms by which J4 increasing the GluR2 expression and whether AMPARs are affected are unclear, and further investigation is required to elucidate the anti-seizure effect of J4 in glutamatergic synaptic transmission.

Our results also revealed a reduced number of DCX- positive cells after J4 pretreatment, while the density of Ki67-positive cells remained unchanged (Fig. 6E-G). This suggests that J4 does not affect cellular proliferation but impacts the differentiation or maturation phase of neurogenesis. The decreased number of DCX-positive cells observed in the J4-treated group may reflect immature neurons shifting toward migration and maturation, suggesting that J4 could facilitate neuronal maturation while not affecting neural precursor proliferation. Alternatively, the reduction in DCX-positive cells may also indicate that J4 limits the differentiation of neural progenitors into immature neurons. Further lineage-tracing studies and analyses of maturation markers will be necessary to clarify these possibilities and the role of J4 in seizure-induced neurogenesis.

J4 has been shown to elevate the extracellular adenosine level in the brain through inhibiting the ENT1 [36, 40]. Herein, we proposed that J4 mitigates seizure severity and reduces excitatory neuronal activities in *Tsc2*^+/–^ mice through adenosine augmentation by ENT1 inhibition, and it is therapeutically promising in the drug-resistant TSC-associated epilepsy. However, the direct measurements of adenosine levels in our experimental conditions were not performed. It would be of great interest to quantify extracellular adenosine changes upon J4 treatment in *Tsc2*^+/–^ mice after PTZ induction to strengthen the mechanistic insights of this novel compound.

In addition to adenosine signaling pathway, one of the possible mechanisms of which J4 reduces the seizure susceptibility of *Tsc2*^+/–^ mice is its anti-inflammatory actions. J4 has been shown previously to block the activation of reactive astrocytes and microglia in a mouse model of Alzheimer’s disease [37]. Likewise, in the PTZ kindling model and PTZ-injected *Tsc2*^+/–^ mice, we also observed that J4 effectively suppressed microglial and astroglial activation resulted from PTZ-induced insults (Fig. 7). Neuroinflammation has been known to strongly associate with the development and progression of epilepsy [66, 67], including TSC-associated epilepsy [68, 69]. In line with the past reports [70–72], our previous study has confirmed that *Tsc2*^+/–^ mice showed astroglial activation when compared to the WT mice [73]. Reactive astrocytes and activated microglia are known to cause neuronal hyperexcitability and excitotoxicity through a various different mechanisms, such as impairments in potassium homeostasis, reduced glutamate uptake, enhanced expression of glutamate receptors, would predispose to seizures [74]. In addition, inflammatory molecules, such as cytokines, chemokines, and complement molecules, released by the activated astrocytes and microglia induce both transcriptional and post-transcriptional events which drive neuronal activity and worsen the excitability [74, 75]. Many studies suggest that neuroinflammation precedes the onset of epileptogenesis and neurodegeneration in several seizure models [76]. In the PTZ-injected *Tsc2*^+/–^ mice, pretreatment of J4, via reducing the activation of reactive astrocytes and activated microglia, moderates the excitation/inhibition balance of the synaptic network in *Tsc2*^+/–^ mice. However, expression of pro-inflammatory cytokines such as IL-1β, TNF-α, and IL-6 should be assessed in future studies to further substantiate J4’s anti-inflammatory effects.

## Conclusion

Based on our results, J4 treatment showed efficacy on reducing the hypersensitivity to the PTZ induction at a sub-convulsant dose in *Tsc2*^*+/–*^ mice. J4 pretreatment increased the expression of GluR2, decreased mossy fiber sprouting, and inhibited the astrogliosis and microgliosis after the PTZ injurious insult, eventually prevented the neuronal cell loss due to the excitotoxicity. Thus, the present study provides a new alternative therapeutic concept using a ENT1 inhibitor, J4 for pretreating TSC-related epilepsy before the initiation of epileptogenesis.

## Supplementary Information

Below is the link to the electronic supplementary material.

Supplementary Material 1

Supplementary Material 2

Supplementary Material 3

Supplementary Material 4

## Data Availability

The data that support the findings of this study are available on request to the corresponding author.
