# Supplementary figures and images for: Anti-seizure potential of J4, an equilibrative nucleoside transporter 1 inhibitor, in a mouse model of tuberous sclerosis complex in response to pentylenetetrazol
Source: Cell Biosci. 2026 Jan 3;16:12. doi: 10.1186/s13578-025-01518-3 (PMC12866362; doi:10.1186/s13578-025-01518-3)

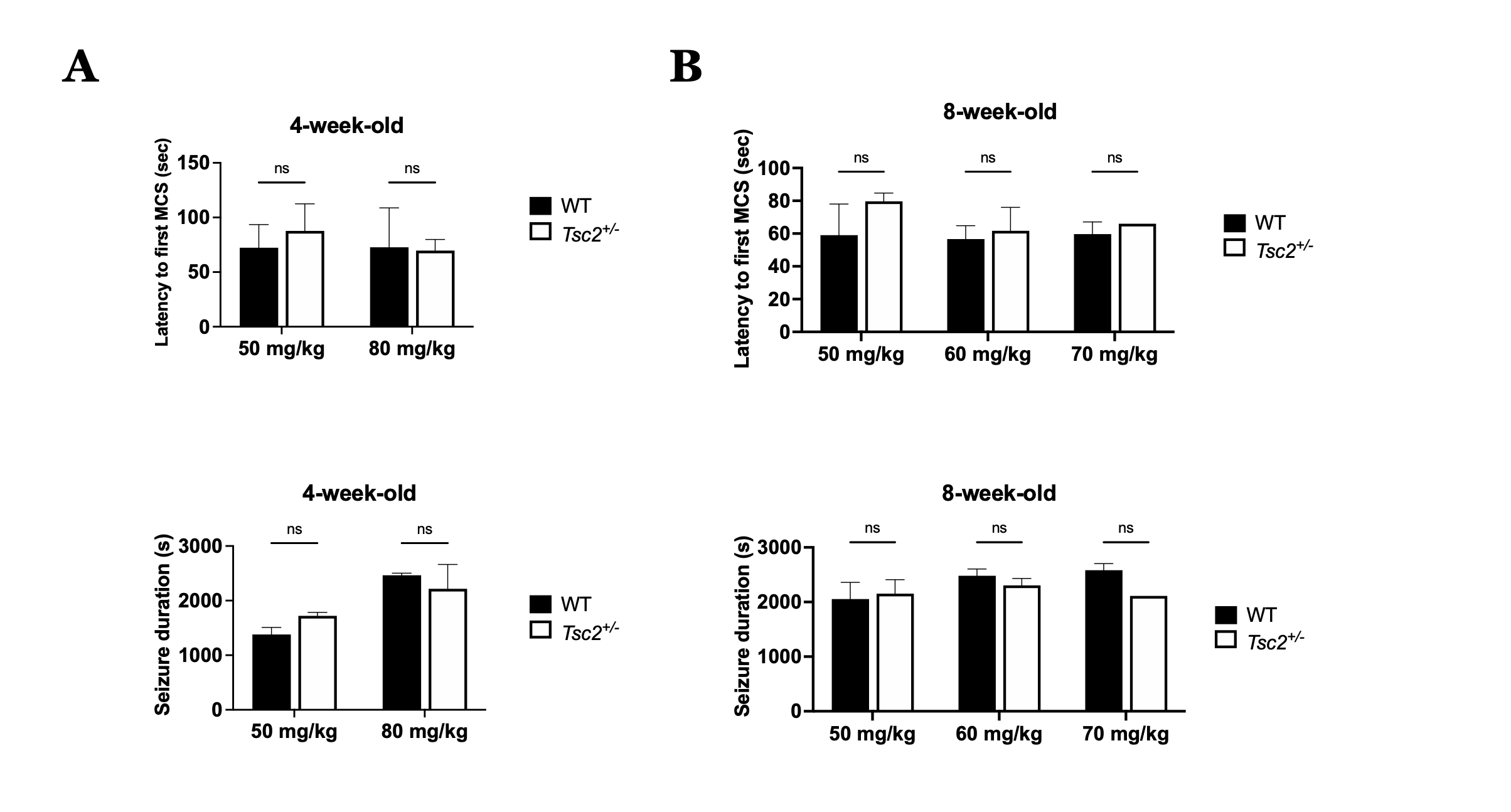

Supplement: Supplementary file 1 — Supplementary Material 1 [file 13578_2025_1518_MOESM1_ESM.tiff]

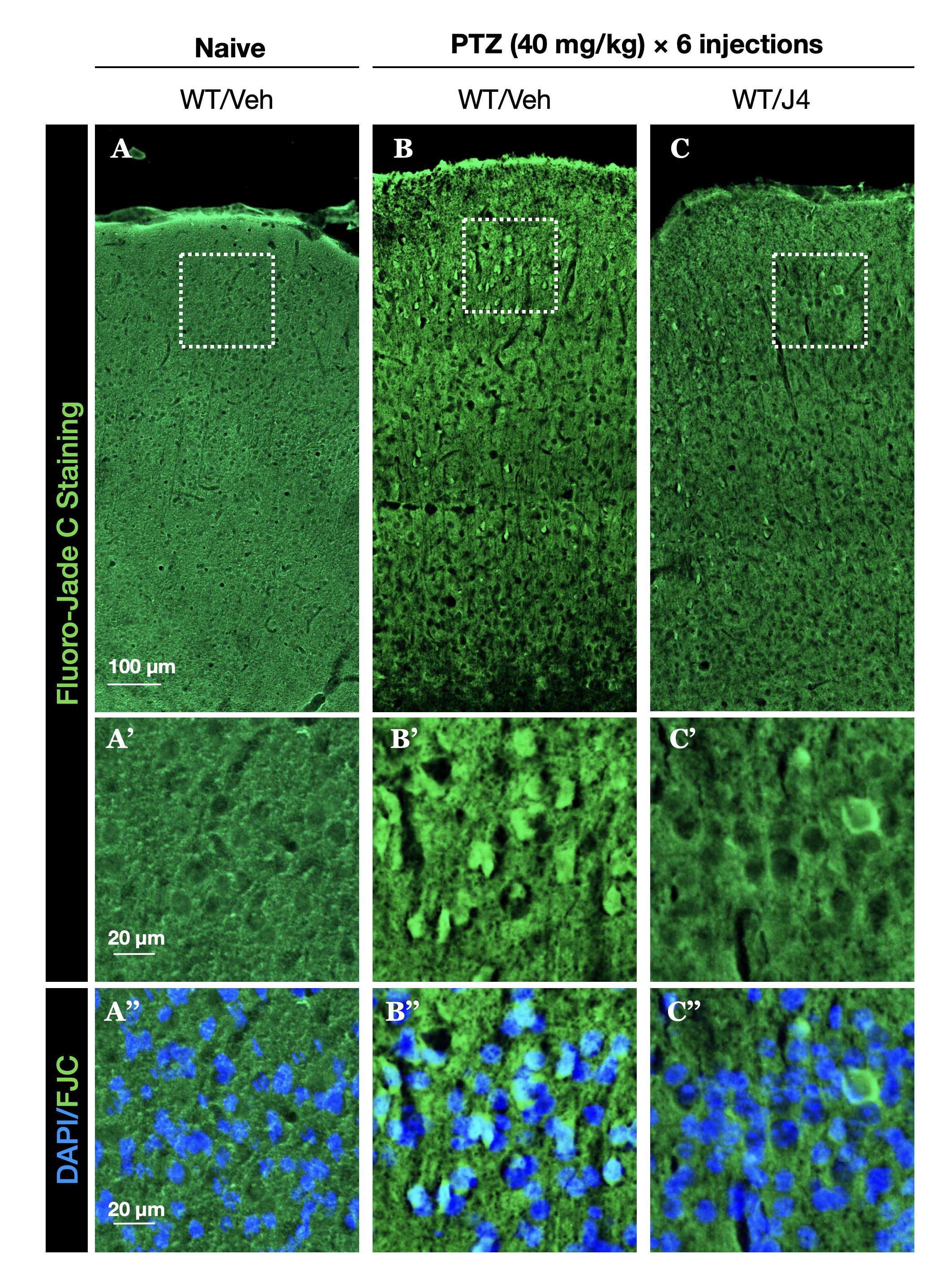

Supplement: Supplementary file 2 — Supplementary Material 2 [file 13578_2025_1518_MOESM2_ESM.tiff]

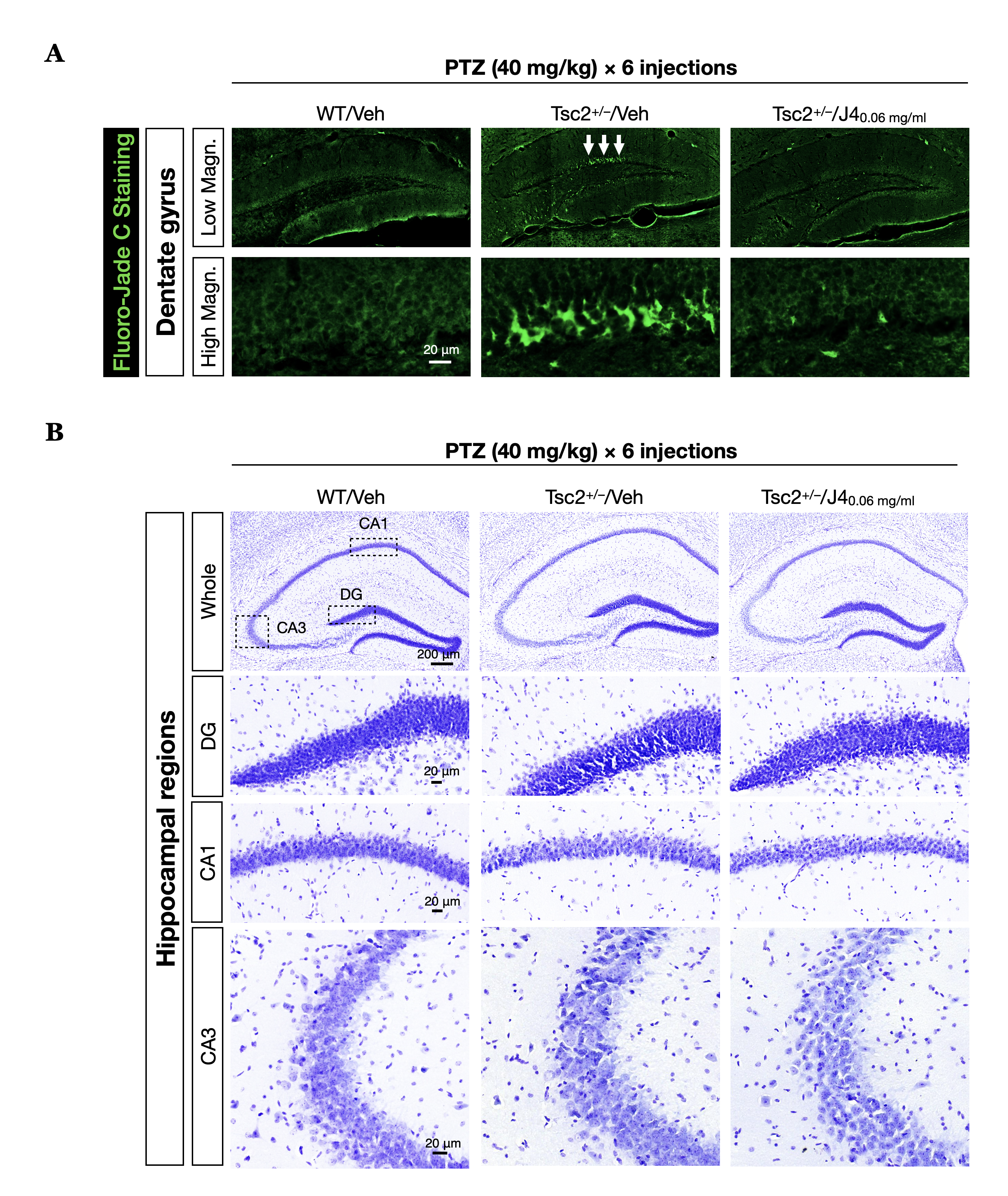

Supplement: Supplementary file 3 — Supplementary Material 3 [file 13578_2025_1518_MOESM3_ESM.tiff]
