# Supplementary material for: Anti-seizure potential of J4, an equilibrative nucleoside transporter 1 inhibitor, in a mouse model of tuberous sclerosis complex in response to pentylenetetrazol
Source: Cell Biosci. 2026 Jan 3;16:12. doi: 10.1186/s13578-025-01518-3 (PMC12866362; doi:10.1186/s13578-025-01518-3)
Supplement: Supplementary file 4 — Supplementary Material 4 [file 13578_2025_1518_MOESM4_ESM.docx]

**Supporting information Figure legends**

**Supplementary Figure 1**

*Tsc2^+/–^ mice showed no differences when injected with high-doses of PTZ*

Different doses of PTZ administration was tested to determine the vulnerability of *Tsc2^+/–^* mice to PTZ. A single intraperitoneal injection of PTZ at high doses (50, 60, 70 and 80 mg/kg) induced an acute, severe seizure behavior in both WT and *Tsc2^+/–^* mice, regardless of the age of the mice. All mice reached Racine score ≥ 4. Latency to first minimal clonic seizure (MCS) and the total time duration of seizures for each animal were shown. *Tsc2^+/–^* mice did not show significant differences at 4-week-old in the latency to first MCS (**Supplemental Fig. 1A, *upper panel***) and the total during time of seizures (**Supplemental Fig. 1A, *lower panel***), when compared to WT mice. Likewise, the two groups did not differ at the age of 8 weeks as well (**Supplemental Fig. 1B**).

**Supplementary Figure 2**

*J4 decreased the number of degenerating cells after PTZ-induced seizures*

Fluoro-Jade C (FJC) staining was performed to analyze the degenerating cells at the entorhinal cortex compared between WT mice without PTZ injections (WT/Veh) (**Supplemental Fig. 2A**) and PTZ-injected WT mice (PTZ-WT/Veh) (**Supplemental Fig. 2B**). The number of FJC-positive cells decreased after pretreatment of J4 (**Supplemental Fig. 2C**).

**Supplementary Figure 3**

*PTZ-induced seizures caused no prominent difference in the dentate gyrus and CA3 of Tsc2^+/–^ mice*

Fluoro-Jade C (FJC) staining was performed to analyze the degenerating cells at the dentate gyrus (**Supplementary Fig. 3A**). After PTZ induced seizures, *Tsc2^+/–^* mice showed increased FJC-positive cells (marked by the arrows) (**Supplementary Fig. 3A**, *middle panel*). J4 pretreatment reduced the FJC-positive cells (**Supplementary Fig. 3A**, *right panel*). Nissl-staining was performed to assess the neuronal loss at the hippocampal subfields, ie., DG, CA1, and CA3 (**Supplementary Fig. 3B**). No prominent changes were observed.
